# Evaluating bacterial community structures in oil collected from the sea surface and sediment in the northern Gulf of Mexico after the *Deepwater Horizon* oil spill

**DOI:** 10.1002/mbo3.89

**Published:** 2013-04-09

**Authors:** Zhanfei Liu, Jiqing Liu

**Affiliations:** Marine Science Institute, The University of Texas at AustinPort Aransas, Texas, 78373

**Keywords:** *Alphaproteobacteria*, bacteria community structure, biodegradation, *Deepwater Horizon* oil spill, *Gammaproteobacteria*, oil mousse, petroleum hydrocarbons, sediment

## Abstract

Bacterial community structures were evaluated in oil samples using culture-independent pyrosequencing, including oil mousses collected on sea surface and salt marshes during the *Deepwater Horizon* oil spill, and oil deposited in sediments adjacent to the wellhead 1 year after the spill. Phylogenetic analysis suggested that *Erythrobacter*, *Rhodovulum*, *Stappia*, and *Thalassospira* of *Alphaproteobacteria* were the prevailing groups in the oil mousses, which may relate to high temperatures and strong irradiance in surface Gulf waters. In the mousse collected from the leaves of *Spartina alterniflora*, *Vibrio* of *Gammaproteobacteria* represented 57% of the total operational taxonomic units, suggesting that this indigenous genus is particularly responsive to the oil contamination in salt marshes. The bacterial communities in oil-contaminated sediments were highly diversified. The relatively high abundance of the *Methylococcus*, *Methylobacter*, *Actinobacteria*, *Firmicutes*, and *Chlorofexi* bacteria resembles those found in certain cold-seep sediments with gas hydrates. Bacterial communities in the overlying water of the oil-contaminated sediment were dominated by *Ralstonia* of *Betaproteobacteria*, which can degrade small aromatics, and *Saccharophagus degradans* of *Gammaproteobacteria*, a cellulose degrader, suggesting that overlying water was affected by the oil-contaminated sediments, possibly due to the dissolution of small aromatics and biosurfactants produced during biodegradation. Overall, these results provided key information needed to evaluate oil degradation in the region and develop future bioremediation strategies.

## Introduction

As the largest marine oil spill in the United States petroleum industry, the *Deepwater Horizon* (*DWH*) oil spill released ∼4.9 million barrels of crude oil to the northern Gulf of Mexico from April to July 2010 (McNutt et al. 2012; Ryerson et al. 2012). The released oil rose to sea surface, stayed in the water column temporarily as the deepwater oil plume, or settled down to the sediment (Camilli et al. 2010; Kessler et al. 2011; Reddy et al. 2011; Ryerson et al. 2011; Liu et al. 2012). Weathering processes, such as spreading, evaporation, dissolution, biodegradation, and photo-oxidation, play a key role in removing the oil from the water, and these processes are effective in different time scales (Whittle et al. 1982; Hazen et al. 2010). Biodegradation processes degrade the oil continuously over time intervals ranging from days to years (Atlas and Hazen 2011; Kessler et al. 2011). It is important to understand how the bacterial communities respond to oil spills to evaluate the resiliency of affected ecosystems and develop future bioremediation strategies.

The *DWH* oil was a light Louisiana crude (API 35.2) that is likely more biodegradable than heavy crude as occurred in the *Exxon Valdez* (Atlas and Hazen 2011). The *DWH* oil spill was distinct from previous spills in that it released not only oil but also a large amount of gases (Camilli et al. 2010; Joye et al. 2011; Kessler et al. 2011), and it occurred at ∼1500 m in the relatively deep ocean. The oil moved across a large spatial scale and was exposed to different environmental conditions, including: deep waters and sediments with low temperature and high pressure, surface waters with high temperature and strong irradiance, and eutrophic salt marsh systems or sandy beaches along the coastline of the northern Gulf of Mexico (Operational Science Advisory Team 2010; McNutt et al. 2012; Mendelssohn et al. 2012; Ryerson et al. 2012). Therefore, the response of bacterial communities in the oil may have depended on environmental conditions. For example, bacterial communities in the deepwater oil plume were dominated by groups of *Oceanospirillales*, *Colwellia*, and *Cycloclasticus* of *Gammaproteobacteria* (Hazen et al. 2010; Valentine et al. 2010; Kessler et al. 2011; Lu et al. 2012; Mason et al. 2012), whereas these groups accounted for only <5% in surface oil sheens, which were dominated instead by *Cyanobacteria* and *Alphaproteobacteria* (SAR11 Clade, *Rhodobacterales*, and *Rhodospirillales*) (Redmond and Valentine 2011). Identified organisms in bacterial community in sandy beach ecosystems include the known oil-degrading genera of *Gammaproteobacteria*, such as *Marinobacter*, *Alcanivorax*, *Pseudomonas*, and *Acinetobacter*, from the oiled sands after the *DWH* oil spill (Kostka et al. 2011). The hydrocarbon-degrading bacteria (*Proteobacteria*, *Bacteroidetes*, *Actinobacteria, and Firmicutes*) were identified in salt marsh sediments impacted by the *DWH* oil spill (Beazley et al. 2012). These studies focused on oil recovered from either the deepwater plume or the coastline. It is also crucial to understand how bacterial communities in the oil slicks or mousses have changed during the travel over tens to hundreds of miles from the accident site to the shorelines. Moreover, the response of the bacterial community to oil deposited in the deep sea sediment, where the oil may remain for years under low temperatures, low concentrations of dissolved oxygen, and high pressures, remains unclear.

**Figure 1:**
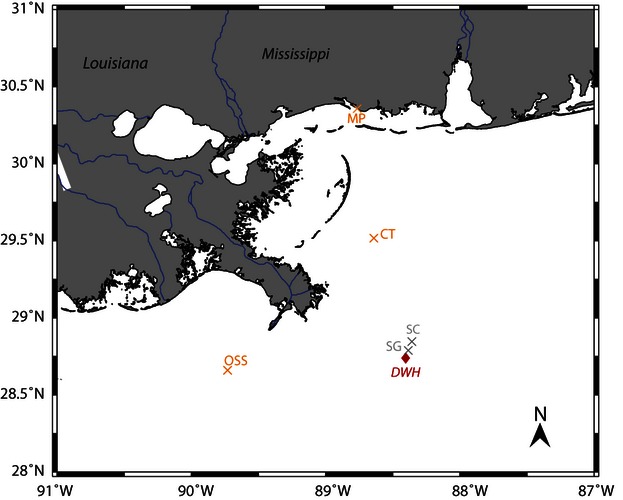
Sampling stations in the northern Gulf of Mexico. Oil mousses and their ambient waters were collected from sea surface at stations OSS and CT, and oil mousse at station MP (Marsh Point, Mississippi). The MP mousse was the most weathered, followed by CT and OSS mousse, based on petroleum hydrocarbon analysis (Liu et al. 2012). Core sediment samples were collected from SG and SC stations. OSS, oil spill site; CT, control; SG, station Grab; SC, station Core.

In this study, we examined bacterial communities in a set of oil samples, including oil mousses collected from sea suface and salt marshes during the oil spill, and oil deposited in sediments adjacent to the wellhead 1 year after the spill (Fig. 1). The hydrocarbon compositions of these oil samples were characterized previously (Liu et al. 2012). The objectives of this study are the following: (1) to decipher bacterial community structures in oil from different environments, including sea surface, salt marshes, and sediments after the *DWH* oil spill, and (2) to examine how bacterial community structures in the oil mousse have changed during the transition due to the weathering process.

## Experimental Procedures

### Sample collection and preparation

#### Oil mousse

Sea surface oil mousses were collected in May 2010 at stations OSS (oil spill site) and CT (control) on board *R/V Pelican* in the northern Gulf of Mexico (Fig. 1). Details of the sample collection were described previously (Liu et al. 2012). Briefly, oil mousse was collected by bucket, and the oil was scraped into acid-cleaned polyethylene bottles. Oil mousse from station CT was light brown in color, while that from station OSS was dark brown with a strong odor. The oil mousse washing onto salt marshes was collected at Marsh Point (MP), Mississippi, on 21 July 2010. The mousse was well emulsified, with 40% of the mass as water (Liu et al. 2012). All of the mousse samples were sealed and stored in a freezer (−20°C) until analysis.

Two surface water samples without visible oil were also collected at stations OSS (OSS-W) and CT (CT-W) during the cruise (Fig. 1). About 2–2.5 L of surface water (∼top 5 cm) was collected using a polypropylene bucket. The water was filtered immediately through pre-combusted 47-mm 0.7 μm GF/F filters (Whatman, Buckinghamshire, U.K.) in a glass filtration apparatus. The filters were stored in a freezer (−20°C) until analysis.

#### Sediments and overlying water

Two sediment samples were collected in May 2011 at stations Grab (28°49.69′N, 88°20.22′W) (SG) and Core (28°50.30′N, 88°19.93′W) (SC), ∼2 and 6 km away from the wellhead, respectively (Fig. 1) (details in Liu et al. 2012). Sediment at SG was collected using a Ponar grab sampler and the surface sediment was scraped using a pre-cleaned stainless steel spoon and transferred into pre-combusted glass jars. Sediment at SC was collected by a sediment corer (Gardner et al. 2009), and the 0–2 cm layer of the SC sediment was sectioned. Oil contamination was observed visually in both sediment samples. About 250 mL of overlying water, within ∼5 cm above the sediment-water interface, was collected from the SC core sample (SC-OW). The overlying water was filtered onto polycarbonate filters (0.2 μm, 47 mm diameter, Poretics). All samples were stored frozen at -20°C until further analysis for bacterial community structure.

### Nucleic acid extraction and DNA fingerprinting

#### DNA extraction

The DNA extraction from oil mousses (0.7–1 g), sediments (0.2–0.4 g) and filters was conducted at the Research and Testing Laboratory (Lubbock, TX) according to the established bacterial DNA extraction procedures (Smith et al. 2010). Briefly, 2 mL of RLT buffer (Qiagen, Valencia, CA) (with β-mercaptoethanol) was added to each sample after the samples were thawed. The samples were centrifuged at 14,000 rpm for 3 min and resuspended in 500 μL RLT buffer. A sterile 5 mm steel bead (Qiagen) and 500 μL sterile 0.1 mm glass beads (Scientific Industries, Inc., NY) were added for complete bacterial lyses in a Qiagen TissueLyser (Qiagen), run at 30 Hz for 5 min. A quantity of 100 μL of absolute ethanol was added to a 100-μL aliquot of the sample supernatant after the samples were centrifuged. The mixture was added to a DNA spin column, and DNA recovery protocols were followed as instructed in the QIAamp DNA Mini Kit (Qiagen) starting at step 5 of the Tissue Protocol. DNA was eluted from the column with 30 μL water. The DNA samples were quantified and measured using a Nanodrop spectrophotometer (Nyxor Biotech, Paris, France) according to the manufacturer's instructions.

#### The bacterial tag-encoded FLX amplicon pyrosequencing (bTEFAP)

Bacterial diversity was studied via the bacterial tag-encoded FLX amplicon pyrosequencing (bTEFAP) using eubacterial primers 28F 5′TTTGATCNTGGCTCAG-3′ and 519R 5′-GTNTTACNGCGGCKGCTG-3′ to amplify the 500 bp region of 16S rRNA gene (Dowd et al. 2008; Smith et al. 2010). Tag-encoded FLX amplicon pyrosequencing analyses utilized a Roche 454 FLX instrument with Titanium reagents. Titanium procedures were performed at the Research and Testing Laboratory according to the RTL protocols (http://www.researchandtesting.com) for bacterial diversity (Smith et al. 2010).

#### Bacterial diversity data analysis and identification

Sequences were analyzed and annotated using a custom scripted bioinformatics pipeline (Handl et al. 2011; Ishak et al. 2011; details can be found at http://www.researchandtesting.com/docs/Data_Analysis_Methodology.pdf). Briefly, short reads < 250 bp were depleted and denoising and chimera checking were accomplished using the UCLUST and UCHIIME algorithms (Edgar 2010; Edgar et al. 2011). To determine the identity of each remaining sequence, the sequences were clustered into operational taxonomic unit (OTU) clusters. For each cluster, the seed sequence was put into a FASTA formatted sequence file. This file was then queried against a highly curated database (compiled by Research and Testing Laboratory) originating from NCBI (http://nbci.nlm.nih.gov) and bacterial taxa were identified using Krakenblast (http://www.krakenblast.com). Based upon BLASTn+ sequence identity, each bacterium was identified to its closest relative and taxonomic level. After best hit processing, genus and higher level taxonomic designations were compiled using a secondary post-processing algorithm and relative percentages of bacterial taxa were determined for each individual sample. The rarefaction curves of all of our samples indicated that our sampling and sequencing efforts covered the extent of taxonomic diversity at 95% confidence level adequately (Figs. S1 and S2).

### Statistical analysis

We applied cluster analysis on Bray-Curtis similarity, using the bacterial community data on the basis of presence or absence of each bacterial genus (307 species) (Bray and Curtis 1957; Liu et al. 2011). The bacteria species that did not occur in any of the samples were not included in the data matrix. With this analysis, the relative percentages of bacteria species in the community were not considered, as the community compositions of these samples differed greatly from each other. This approach is particularly useful for samples with a large number of variables (Sleighter et al. 2010; Liu et al., 2011).

## Results

### Bacterial community structures in oil mousses and their ambient waters

Oil mousses were collected from sea surface at stations OSS and CT, and from leaves of *Spartina alterniflora* at station MP (Fig. 1). The pyrosequencing analysis on the oil mousses from stations OSS, CT, and MP yielded 3173, 86, and 2575 distinct bacterial OTUs, respectively; 86, 85, and 54 genera of bacteria from four to five phyla were determined from these three mousses accordingly (Table 1). In contrast, 6707 and 5775 OTUs were determined from OSS and CT ambient surface waters (OSS-W, CT-W) without visual oil contamination, of which 51 and 52 genera of bacteria (six and seven phyla) were identified, respectively.

**Table 1:** Total relative abundances of main bacterial groups from pyrosequencing

Sample	OSS-W	CT-W	SC-OW	SG	SC	OSS	CT	MP
Total OTUs	6707	5775	3125	668	577	3173	1986	2575
Total genera	51	52	35	111	95	86	85	54
Dominant genera (≥1%)	4	4	15	25	30	25	17	10
*Alphaproteobacteria* (%)	1.9	2.3	13.3	26.2	19.1	74.9	65.5	15.6
*Gammaproteobacteria* (%)	0.0	0.0	21.1	16.3	33.6	21.0	27.7	75.8
Other proteobacteria (%)	0.1	0.0	53.2	21.6	16.6	0.1	2.0	7.8
*Flavobacteriales* (%)	0.0	0.0	2.8	1.0	2.6	3.2	2.5	0.2
*Planctomycetes* (%)	0.9	1.4	1.6	5.8	2.4	0.0	0.0	0.0
*Bacteroidetes* (%)	0.0	0.1	2.8	2.1	6.1	3.3	2.9	0.2
*Firmicutes* (%)	0.1	0.5	1.1	6.4	3.8	0.0	1.3	0.0
*Actinobacteria* (%)	0.4	1.5	0.0	7.2	8.1	0.2	0.7	0.3
*Chloroflexi* (%)	0.0	0.0	0.0	3.7	3.5	0.0	0.0	0.2
*Cyanobacteria* (%)	96.6	94.0	0.2	0.3	0.2	0.5	0.0	0.2
Other (%)	0.0	0.1	0.4	6.0	2.9	0.1	0.0	0.0
Shannon indices	2.1	1.8	4.4	9.8	8.6	2.0	2.6	2.1

In May 2010, oil mousses were collected from sea surface at stations OSS, CT, and from salt marsh at Marsh Point (MP), Mississippi. Ambient water without visual oil contamination was also collected at station OSS and CT (OSS-W and CT-W). Sediments were collected in May 2011 at stations SG and SC, and the overlying water of SC sediments (SC-OW) was also collected. OSS, oil spill site; CT, control; SG, station Grab; SC, station Core; OTUs, operational taxonomic units.

**Figure 2:**
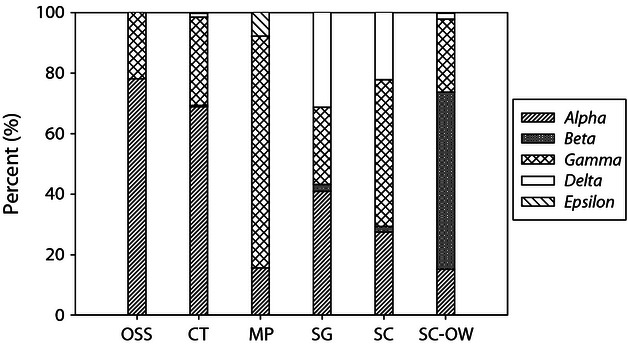
Relative percentages of different classes of *Proteobacteria* in oil mousses (OSS, CT, and MP), sediments (SG and SC), and overlying waters of SC sediment (SC-OW). OSS, oil spill site; CT, control; MP, Marsh Point; SG, station Grab; SC, station Core.

Phylogenetic analysis showed that *Proteobacteria* was the most dominant group (phylum) in the three oil mousses, ranging from 95% to 99% of the total (Table 1). In contrast, members of *Proteobacteria* in OSS and CT ambient waters represented only 2% of the OTUs, while the dominant bacterial group was *Cyanobacteria* at 94%. The significant difference of bacterial community structures between oil mousse and ambient waters suggested that bacteria in the oil mousse did not accumulate simply from the water, but developed through involvement in oil degradation. Within the *Proteobacteria, Alpha* and *Gamma* classes together dominated the oil mousse sequencing and the two ambient waters, ranging from 92.1% to 99.9% (Table 1 and Fig. 2). *Bacteroidetes* and *Actinobacteria* occurred in all mousse samples and ambient waters. *Flavobacteria* appeared only in oil mousse samples, but were more abundant in OSS and CT than MP. *Firmicutes* were present in all samples except for OSS and MP oil mousses (Table 1).

**Figure 3:**
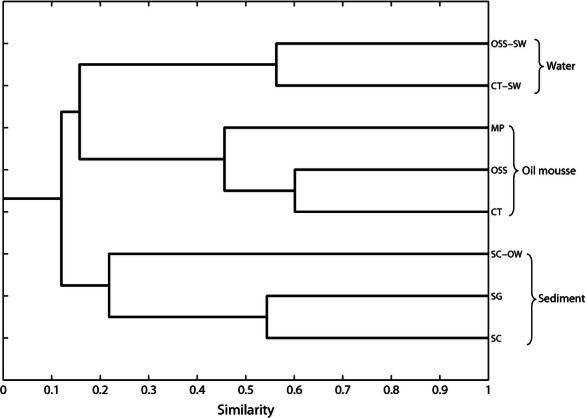
Bray-Curtis analysis on bacterial communities based on absence or presence of each bacterial species in oil mousses (OSS, CT and MP), sediments (SG and SC), and overlying waters of SC sediment (SC-OW). OSS, oil spill site; CT, control; MP, Marsh Point; SG, station Grab; SC, station Core.

**Figure 4:**
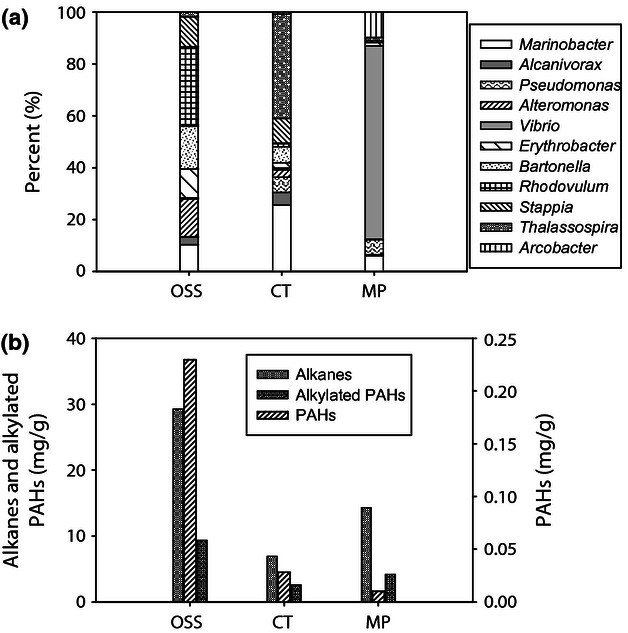
(a) Compositions of main oil-degraders in the classes of *Alpha-* and *Gamma-proteobacteria* in oil mousses collected at stations OSS, CT and MP, except for *Arcobacter*, which belongs to *Epsilonproteobacteria*; (b) Concentrations of petroleum hydrocarbons in three oil mousses, including total alkanes (C_9_-C_38_, pristane, and phytane), 16 polycyclic aromatic hydrocarbons (PAHs), and 18 alkylated PAHs (data based on Liu et al. 2012). OSS, oil spill site; CT, control; MP, Marsh Point.

Cluster analysis showed that bacteria communities of OSS and CT mousses were closer to each other than to those in the MP mousse, but all of the three mousses were distinctly different from those of the surface water communities (Fig. 3). Despite the similarity between OSS and CT oil mousses, the relative abundance of each group of bacteria differed from each other. For example, *Thalassospira* and *Rhodovulum* represented 24% and 1% of the total *Alpha*- and *Gammaproteobacteria* OTUs in the CT oil mousse, whereas they accounted for 1% and 17% in the OSS oil mousse, respectively (Fig. 4). In the MP mousse, *Vibrio* represented 57% of total bacterial abundance, but constituted less than 1% or was undetected in other mousses. In addition, *Arcobacter* represented 7.8% of total OTUs in MP mousse, much higher than that in OSS (undetected) and CT (1%) mousses.

The major well-documented aerobic hydrocarbon-degrading bacteria including genera *Marinobacter*, *Alcanivorax*, *Pseudomonas*, *Alteromonas* (Head et al. 2006; Yakimov et al. 2007; Hazen et al. 2010; Redmond and Valentine 2011), occurred in CT, OSS, and MP oil mousses, and these bacteria were the dominated groups within *Gammaproteobacteria*, representing 82.7%, 74.7%, and 13.0%, respectively (Fig. 4 and Table S1). The genus *Vibrio*, often abundant in highly entrophic coastal ecosystems, was the dominant bacteria in MP oil mousse, representing 79% of the total *Gammaproteobacteria*. The *Alcanivorax* group appeared with 56, 54, and 10 OTUs in CT, OSS, and MP mousses, respectively. *Alcanivorax* may be obligate degraders of alkanes, and often dominate the bacterial community in seawater at the initial stage of oil degradation (Harayama et al. 2004). *Alphaproteobacteria* was the dominant group in CT and OSS oil mousses, except for the MP oil mousse dominated by *Gammaproteobacteria* (Fig. 2). *Erythrobacter*, *Bartonella*, *Rhodovulum*, *Stappia*, and *Thalassospira* were the dominant genera in the three oil mousses, constituting 54%, 54%, and 83% of total *Alphaproteobacteria*, respectively (Fig. 4 and Table S2). The dominance of these bacterial species suggests that they may play an important role in degrading polycyclic aromatic hydrocarbons (PAHs) (Röling et al. 2004; Coulon et al. 2007; Kodama et al. 2008; Teramoto et al. 2010; Beazley et al. 2012). For example, *Thalassospira* bacteria, the newly found member of *Alphaproteobacteria*, can degrade multiple types of PAHs (Kodama et al. 2008). Consistently, *Thalassospira* occurred in oil mousses, especially in CT oil mousse accounting for 36% of total *Alphaproteobacteria* (Fig. 4).

### Bacterial community in sediment and sediment overlying water

The pyrosequencing identified diverse bacterial groups in sediments from stations SC and SG, with 111 and 95 genera from 16 and 14 phyla, respectively (Table 1). In comparison, bacteria in the overlying water of SC sediment (SC-OW) were much less diverse, with 35 genera from 3125 OTUs. The bacterial communities in SC and SG sediments consisted mainly of *proteobacteria*, accounting for 64.1% and 69.3% of total bacteria, respectively (Table 1). *Gamma-*and *Alphaproteobacteria* together accounted for a major fraction of the *proteobacteria* in SC (42.5%) and SG (52.7%) sediments, while the *Betaproteobacteria* represented only 1.2–1.5% (Fig. 2). In the SC overlying water, *proteobateria* represented 87.6% of total OTUs (1601), of which *Betaproteobacteria* accounted for 51.2%, and *Gamma-* and *Alphaproteobacteria* together represented 34.4%. In addition, *Bacteroidetes, Actinobacteria*, and *Planctomycetes* occurred in all sediments and overlying water. Cluster anaysis showed that bacterial community structures of SC and SG sediments were similar, but those of the SC overlying water were different than the sediment communities (Fig. 3).

**Figure 5:**
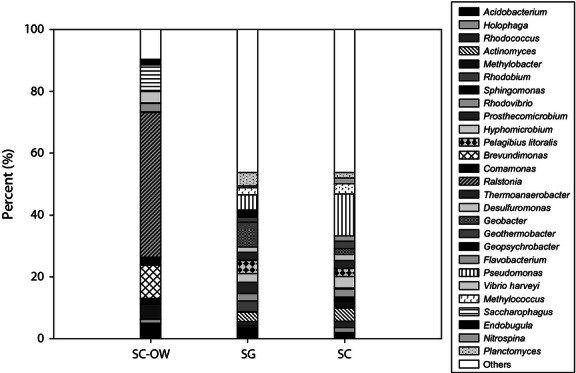
Compositions of bacterial communities in sediments collected at stations SG and SC, and in overlying waters of SC sediments (SC-OW). The relative abundance of each bacterial species shown in the figure was at least 2% or above in one of the three samples. The species not shown on the bar were categorized as others. Clearly, bacterial communities in the SG and SC sediments were much more diverse than those of the SC-OW. SG, station Grab; SC, station Core; SC-OW, overlying water of SC sediment.

*Alcanivorax* and *Pseudomonas* were detected in sediments from both sites, indicating that these bacteria were involved in the oil degradation (Head et al. 2006; Yakimov et al. 2007). The *Oceanospirillales*, *Colwellia*, and *Cycloclasticus* groups, which dominated the deepwater oil plume during the *DWH* oil spill (Hazen et al. 2010; Valentine et al. 2010; Redmond and Valentine 2011; Mason et al. 2012), appeared in both sediments and overlying waters, but only in trace quantities (Tables S1 and S2). For example, 2–8 OTUs of *Colwellia* were identified in sediments, and 4 OTUs of *Cycloclasticus* in the overlying water of SC sediment. Instead, microbial communities in sediments included mainly *Methylococcus* (*methylotrophs*), *Vibiro*, *Pseudomonas* of *Gammaproteobacteria*, *Methylobacterium* (*methanotrophs*) of *Alphaproteobacteria, Flavobacteria*, and *Acidobacteria* (Fig. 5), suggesting that these taxa were important in hydrocarbon degradation. For example, some members of the genus *Pseudomonas* can metabolize a variety of simple aromatic organic compounds and PAHs (Arun et al. 2011). Also, *Rhodococcus*, which appeared in both sediment samples (Fig. 5), can degrade aromatics (Sorkhoh et al. 1990). In addition, *Flavobacteria* were detected in sediments and overlying waters; these bacteria, found commonly in the ocean, are associated with the degradation of high-molecular-weight disssolved organic matter (Cottrell and Kirchman 2000; Kirchman 2002).

## Discussion

### The evolution of bacterial community structure in oil mousses

After the *DWH* oil spill, studies on bacterial community structures and the active oil degraders have focused mostly on the deepwater oil plume (Hazen et al. 2010; Valentine et al. 2010; Kessler et al. 2011; Redmond and Valentine 2011; Lu et al. 2012; Mason et al. 2012), and oil in sandy beaches or salt marshes (Kostka et al. 2011; Beazley et al. 2012). But an understanding of how bacterial communities evolve in the surface oil during transport from the accident site to salt marshes along the coast remains unclear.

*Oceanospirillales*, *Colwellia*, and *Cycloclasticus* of *Gammaproteobacteria* dominated the bacterial community in the deepwater oil plume (Hazen et al. 2010; Valentine et al. 2010; Kessler et al. 2011; Redmond and Valentine 2011; Bælum et al. 2012; Lu et al. 2012; Mason et al. 2012). Once oil rose to sea surface (2 km from the accident site), the bacterial community in the oil slick was dominated by 100% of *Gammaproteobacteria* (93% *Pseudoalteromonas*), but its percentage decreased to 48% (*Pseudomonas*, *Vibrio*, *Acinetobacter*, *and Alteromonas*) in oil collected at a station further from the accident site (44 km), along with ∼30% of *Alphaproteobacteria* (*Bacteroidetes* and SAR 11); temperature fluctuation was attributed as the factor leading to this community shift from deepwater (4°C) to sea surface (20°C) (Redmond and Valentine 2011). Our data showed that *Alphaproteobacteria* continued to increase to over 60% in the oil mousses collected at stations OSS and CT, 135 and 85 km away from the accident site. Petroleum hydrocarbon analysis suggested that OSS and CT mousses were subjected to moderate weathering, as low-molecular-weight hydro carbons such as *n*-alkanes (*n* < 15) and naphthalene homologues dissapeared due to evaporation (Reddy et al. 2011; Liu et al. 2012). Together these data suggest that *Alphaproteobacteria* became more dominant as the surface oil was weathered gradually during the movement, consistent with laboratory incubation results indicating that *Alphaproteobacteria* become dominant at the later stage of oil degradation (Röling et al. 2004; Beazley et al. 2012).

*Gamma-* and *Alphaproteobacteria* dominated the bacterial communties in both OSS and CT mousses (>90%). The community structures of the two oil mousses were similar in *Gammaproteobacteria*, but remarkably different in *Alphaproteobacteria*, which may relate to the hydro carbon composition of the mousse (Fig. 4). The occurrences of *Alcanivorax* (2–3% OTUs), which are excellent degraders of alkanes including branched ones (Hara et al. 2003; Head et al. 2006), in both OSS and CT oil mousses suggest that these bacteria were degrading alkanes. This argument is supported by the lower ratios of *n*-C_17_/pristane and *n*-C_18_/phytane and lower concentrations of total alkanes in the CT mousse than the OSS mousse (Fig. 4 and Table 2). *Marinobacter and Alteromonas*, which are common oil-degrading genera of *Gammaproteobacteria* (Head et al. 2006), also occurred in the two mousses, representing 15% OTUs. These genera can use a broad range of carbon substrates (Kostka et al. 2011). Consistent with previous work (Redmond and Valentine 2011), *Cycloclasticus* was not detected in the surface mousses, even though this genus is thought to be the main degrader of PAHs (Harayama et al. 2004; Head et al. 2006; Hazen et al. 2010; Bælum et al. 2012), and can grow in both 4°C and 20°C (Coulon et al. 2007). This phenomenon may be explained by high sea surface temperatures at stations OSS (28.7°C) and CT (28.4°C) when samples were collected, which may be too high for *Cycloclasticus* growth. More research is needed to test the optimum temperature range for *Cycloclasticus*.

**Table 2:** Concentrations of petrolume hydrocarbons in oil mousses collected at stations OSS, CT, and MP, and surface sediments at stations SG and SC (data adapted from Liu et al. 2012)

	MC252 crude (mg/g)	OSS (mg/g TSEM)	CT (mg/g TSEM)	MP (mg/g TSEM)	SC (mg/g)	SG (mg/g)
ΣPAHs*	1.15	0.23	0.03	0.01	0.0003	0.0010
Phen/Chry	6.82	1.04	0.28	0.08	2.97	1.06
ΣAlkanes	81.06	29.30	6.91	14.28	0.004	2.56
ΣAlkylated PAHs	7.20	9.36	2.56	4.16	0.03	1.66

Phen/Chry, ratio of phenanthrene over chrysene, an indicator of biodegradation; ΣAlkanes include n-alkanes (C_8_–C_38_), pristane, and phytane; ΣAlkylated PAHs include naphthalene, phenanthrene, fluoranthene, pyrene, chrysene, anthracene, and retene homologues, 18 compounds altogether. The hydrocarbons were normalized to total solvent extractable materials (TSEM) in oil mousses, and to dried weight gram in surface sedimients.

* ΣPAHs include the 16 types of US EPA PAHs.

Belonging to *Alphaproteobacteria*, *Erythrobacter* appeared with 6% OTUs in OSS mousse, but only 1% in CT mousse. *Erythrobacter*, anoxygenic phototrophic bacteria (Kolber et al. 2001), may contribute to aromatic hydrocarbon degradation in oil-contaminated beaches (MacNaughton et al. 1999; Chung and King 2001; Röling et al. 2004; Beazley et al. 2012). *Erythrobacter* grew well in OSS mousse due to strong irradiance on sea surface in the Gulf and ample oil substrates, but its percentage decreased in CT mousse, perhaps caused by further decrease of *n*-alkane and aromatic hydrocarbon contents in the mousse (Fig. 4). Another dominant *Alphaproteobacteria* genus in the OSS mousse was *Rhodovulum*, accounting for 17% of the total OTUs. In tropical waters, two *Rhodovulum-*related strains, which can degrade petroleum aromatics, were identified (Teramoto et al. 2010). As mentioned above, the high surface water temperatures in the northern Gulf of Mexico were optimal for the growth of *Erythrobacter* and *Rhodovulum* (Koblížek et al. 2003; Kumar et al. 2008). *Stappia* of *Rhodobacterales*, representing 6% of total OTUs in both OSS and CT mousses, can degrade both aliphatic and aromatic petroleum hydrocarbons under relatively high temperature (Al-Awadhi et al. 2007; Coulon et al. 2007). High abundance of *Thalassospira* bacteria occurred in the oil mousse, especially in CT oil mousse with 23% of total OTUs. The mesophilic *Thalassospira* can use aromatics, such as naphthalene, dibenzothiophene, phenanthrene, and fluorene, efficiently as substrates (Kodama et al. 2008). The *Rhizobiales* order was also abundant in OSS and CT mousses (10–15% of total OTUs), consisting of genera *Bartonella*, *Methylobacterium*, *Agrobacterium*, and *Parvibaculum*, the degraders of aromatics (Wang et al. 2008). We conclude that the dominance of these *Alphaproteobacteria* in the two mousses may relate to strong irradiance and high temperature in Gulf surface waters.

*Alphaproteobacteria* might be important in degrading the aromatic hydrocarbons, since *Erythrobacter*, *Rhodovulum*, *Stappia*, and *Thalassospira* can degrade aromatics, as described above. Consistently, concentrations of PAHs and alkylated PAHs were four to eight times lower in CT oil mousse than in OSS mousse (Fig. 4), as CT mousse was more weathered than the OSS one (Liu et al. 2012). Moreover, the ratios of phenanthrene/chrysene, an indicator of oil degradation (Pastor et al. 2001), were 1.0, 0.3, and 0.1 in OSS, CT, and MP mousse, respectively (Table 2), supporting the important role of these *Alphaproteobacteria* in degrading aromatic hydrocarbons after the first wave of degradation, when small-chained *n*-alkanes were lost already due to evaporation (Liu et al. 2012). More research on metabolic genes is needed to decipher the direct role of these bacteria in the degradation process.

The bacterial community in MP mousse consisted of some common oil degraders found in OSS and CT mousses, including *Thalassospira*, *Stappia*, *Erythrobacter*, *Alcanivorax*, and *Marinobacter*, as expected. However, two unique genera occurred in the MP mousse. *Arcobacter*, belonging to *Epsilonproteobacteria*, was found in 7.8% of total OTUs, and this genus may relate to sulfide oxidation in oil-contaminated environments under microaerophilic conditions (Voordouw et al. 1996; Gevertz et al. 2000; Wirsen et al. 2002). The relatively high abundance of *Arcobacter* in MP mousse suggests that this marsh system is highly eutrophic, and these bacteria might originate from the suboxic or anoxic sediment and migrate to the oil by sediment resuspension. The *Vibrio* genus was remarkably abundant, representing 57% of the total OTUs in MP mousse. Consistently, the abundance of *Vibrio vulnificus* was 10–100 times higher in tars balls washed onto marshes and beaches along the coast of Mississippi and Alabama after the *DWH* oil spill than that in seawater and sands (Tao et al. 2011). *Vibrio* was identified in surface mousse both near the accident site, sandy beaches, and marsh sediments, but only as a minor component (Kostka et al. 2011; Redmond and Valentine 2011; Beazley et al. 2012). *Vibrio* is often a dominant species in eutrophic salt marsh systems (Ansede et al. 2001). Therefore, the dominance of *Vibrio* in MP mousse but not in the other samples suggests that these indigenous bacteria of salt marshes may degrade aromatic and perhaps other hydrocarbons actively (West et al. 1984; Hedlund and Staley 2001; Thompson et al. 2004; Beazley et al. 2012). This argument is supported by the three-time decrease of total PAH levels from CT to MP mousse (Fig. 4). *Vibrio* could have been simply accumulated in the mousse, but it is more plausible that *Vibrio* became involved in oil degradation to take advantage of the carbon substrate, considering its dominance in the community structure. The bacterial community, including *Proteobacteria*, *Actinobacteria*, *Bacteroidetes*, and *Firmicutes*, was more diversified in sediments of coastal salt marshes impacted by the *DWH* oil spill (Beazley et al. 2012), which resembles those in sediment contaminated by the oil (later) more than those in the MP oil mousse collected from the marsh grass. This finding suggests the influence of indigenous bacterial communities on development of the oil degraders.

### Bacterial community structures in sediments and overlying water

Results from the sediments and overlying waters sampled 1 year after the *DWH* oil spill suggest that the observed bacterial communities were involved with later stages of biodegradation. Bacterial community diversity tends to decrease at the initial stages of biodegradation, followed by an increase at later stages in laboratory petroleum incubations (Röling et al. 2004). The high Shannon indices in SG and SC sediments (Table 1) suggest that the bacterial communities may have recovered partially after the initial oil pulse, as the dominant species *Oceanospirillales*, *Colwellia*, and *Cycloclasticus* in the deepwater plume (Hazen et al. 2010; Redmond and Valentine 2011), constituted only minor percentages in the sediment. It is also possible that bacterial communities in sediments were different and more complex than those in the water column at the beginning of the biodegradation. *Alpha-* and *Gammaproteobacteria* together accounted for 40–50% of the total OTUs, but no species was as dominant as those in the oil mousses. *Pseudomonas* of *Gammaproteobacteria* represented 5% and 14% of the SG and SC communities, respectively. In addition, *Hyphomicrobium* and *Rhodovibrio* of *Alphaproteobacteria* each accounted for about 2% of the sediment bacterial communities, and these species are known to relate to oil contamination in marine environments (Head et al. 2006; Wu et al. 2009; Kostka et al. 2011). Presumably they were degrading oil actively, since considerable oil, including relatively labile low-molecular-weight *n*-alkanes, aromatics and BTEX (benzene, toluene, ethylbenzene, and *p*-, *m-*, and *o*-xylenes), remained in these sediments one year after the spill (Liu et al. 2012).

*Methylococcus* and *Methylobacter* of *Gammaproteobacteria* accounted for 3–7% of the bacterial communities in SG and SC sediments and SC overlying waters. The occurrence of these type I methanotrophs suggests the existence of low concentrations of methane (Hanson and Hanson 1996). A fraction of methane might be trapped in the oil that was deposited in the deep-sea sediment under the low temperature and high pressure after the *DWH* oil spill, considering that over 50% of the crude Macondo oil was methane (Ryerson et al. 2011). In addition, methane may have been produced from anaerobic degradation of petroleum hydrocarbons (Widdel and Rabus 2001). This argument is supported by the presence of 1% *Desulfobacterium* sp., a sulfate-reducing bacterium (Egli et al. 1987), in the bacterial communities of both SG and SC sediments.

A smaller Shannon Index (4.4) suggests that the bacterial community in the overlying water was not as diversified as in the sediments (Table 1). The oil on surface sediment may have affected the bacterial community in the overlying water. For example, the oil degraders *Vibrio harveyi* and *Brevundimonas* sp. accounted for 4% and 11% of the community in the overlying water, respectively (Harwati et al. 2007; Kryachko et al. 2012). *Ralstonia* of *Betaproteobacteria*, which can degrade hydrocarbons (Płaza et al. 2008), dominated the bacterial community (47% of total OTUs). The *Ralstonia* strain can degrade BTEX as the sole carbon source (Lee and Lee 2001). Considering that appreciable quantities of BTEX remained in the sediment (1–2 μg/g) (Liu et al. 2012), perhaps these relatively soluble aromatic may diffuse to the overlying water slowly and be degraded by *Ralstonia*. The excitation-emission matrix (EEM) analysis on the overlying water showed a clear oil-related contour (Ex 270–290 nm, Em 330–360 nm, data not shown), which resembles an oil component (C2) of the crude oil (Zhou et al. 2013). Interestingly, *Saccharophagus degradans*, a cellulose degrader (Zhang and Hutcheson 2011), represented 9% of the bacterial community in the overlying water. One possible explanation for the growth of this bacterium is that oil degraders in sediment produced polysaccharide-related biosurfactants (Desai and Banat 1997), such as rhamnolipids by *Pseudomonas*. Perhaps biosurfactants were degraded by these bacteria after they diffused into the overlying water. More research is warranted for testing this speculation.

Oil seeps are widespread in the Gulf of Mexico, so it is not surprising that indigenous bacteria have a strong potential to degrade hydrocarbons (MacDonald et al. 1989). In methane and sulfate-rich cold seeps, the microbial communities in surface sediments (∼6 cm) were dominated by *Epsilon*- and *Deltaproteobacteria* (Mills et al. 2003; Reed et al. 2006). *Beggiatoa* mat (*Gammaproteobacteria*) can also dominate surface sediments of cold hydrocarbon seeps (Sassen et al. 1993). These bacterial communities differ from those in SG and SC sediments. However, bacterial communities in certain cold oil seeps resemble those in the SG and SC sediments. For example, relatively high abundance of *Pseudomonas* of *Gammaproteobacteria*, *Actinobacteria*, and low G+C *Firmicutes* (33–36% each) were identified at a station with extensive gas hydrates in the northern Gulf of Mexico (Lanoil et al. 2001). Similarly, relatively high percentages of *Actinobacteria* and *Firmicutes* (4–8% each) occurred in SG and SC sediments. *Chlorofexi*/green non-sulfur bacteria were identified in high abundance (12% clones) in the bacterial communities from the cold-seep sediments from Florida Escarpment (Reed et al. 2006). Likewise, we identified about 4% OTUs as *Chlorofexi*, suggesting that bacterial activities in the SG and SC sediments resemble certain oil-seep sediments (Morris et al. 2004).

## Conclusions

The *DWH* oil spill provides a unique opportunity to examine changes in bacterial community structures during oil movement and degradation, as this oil spill occurred across an enormous spatial gradient with drastically different environmental conditions. Environmental conditions and oil weathering are both important factors in controlling the oil degraders in oil mousses. Bacterial communities in two sea surface oil mousses (OSS and CT) differed significantly, even though both were dominated by *Alphaproteobacteria*. *Rhodovulum* dominated the OSS mousse, while *Thalassospira* dominated the CT mousse. This community shift appeared related tightly to oil weathering, as the measured petroleum hydrocarbon concentrations decreased four to eight times from OSS to CT mousse. However, as typical dominant oil-degraders, *Alcanivorax* did not occur in abundance and *Cycloclasticus* was not detected in mousses, demonstrating the unique features of the *DWH* oil spill. High temperature and strong irradiance in the Gulf of Mexico may have played a key role in shaping the bacterial community in the oil mousses. *Vibrio* and *Arcobacter* thrived in the oil mousse collected in salt marshes, suggesting that these indigenous bacteria responded well on the oil contamination and were important degraders of the petroleum hydrocarbons. In contrast to those in mousses, bacterial communities in oil-contaminated sediments were more diverse 1 year after the *DWH* oil spill. In addition to the presence of typical oil degraders, such as *Pseudomonas*, *Hyphomicrobium*, and *Rhodovibrio*, a relative high abundance of methanotrophs was also identified. The bacterial community in the overlying waters of the oil-contaminated sediment was dominated by *Ralstonia* sp. and *Saccharophagus degradans*, suggesting that some light aromatics such as BTEX and biosurfactants might diffuse from sediment into the overlying waters. Overall, this study provides important baseline information for evaluating the role of bacteria in oil removal and developing bioremediation strategies in the northern Gulf of Mexico after oil spills.

## Supporting Information

**Figure S1**. Rarefaction curves of the samples collected in the northern Gulf of Mexico during or after the *Deepwater Horizon* oil spill. The samples included oil mousse collected at stations OSS, CT and MP. Ambient water without visible oil was also collected at station OSS and CT (OSS-W, CT-W). The overlying water was collected at station SC (SC-OW). Rarefaction curves of all samples reached saturation, indicating that our sampling and sequencing efforts adequately covered the extent of taxonomic diversity at 95% confidence level.

**Figure S2.** Rarefaction curves of the sediment samples collected at stations SG and SC in the northern Gulf of Mexico one year after the *Deepwater Horizon* oil spill. Rarefaction curves of both samples reached saturation, indicating that our sampling and sequencing efforts adequately covered the extent of taxonomic diversity at 95% confidence level.

**Table S1**. Identification of known oil degraders in the *Gammaproteobacteria* class. The data showed both operational taxonomic units (OTUs) and the percentages of the bacterial species.

**Table S2**. Identification of known oil degraders in the *Alphaproteobacteria* class. The data showed both operational taxonomic units (OTUs) and the percentages of the bacterial species.
