# Evaluating bacterial community structures in oil collected from the sea surface and sediment in the northern Gulf of Mexico after the deepwater horizon oil spill

**DOI:** 10.1002/mbo3.117

**Published:** 2013-08-12

**Authors:** Zhanfei Liu, Jiqing Liu

***MicrobiologyOpen* 2013; 2(3): 492–504**

On page 496, under the heading “**Bacterial community in sediment and sediment overlying water**”, the terms SC and SG have been wrongly swapped in some places.

The paragraph should read like the following:

The pyrosequencing identified diverse bacterial groups in sediments from stations **SG** and **SC**, with 111 and 95 genera from 16 and 14 phyla, respectively (Table 1). In comparison, bacteria in the overlying water of SC sediment (SC-OW) were much less diverse, with 35 genera from 3125 OTUs. The bacterial communities in **SG** and **SC** sediments consisted mainly of *proteobacteria*, accounting for 64.1% and 69.3% of total bacteria, respectively (Table 1). *Gamma-* and *Alphaproteobacteria* together accounted for a major fraction of the *proteobacteria* in **SG** (42.5%) and **SC** (52.7%) sediments, whereas the *Betaproteobacteria* represented only 1.2–1.5% (Fig. 2). In the SC overlying water, *proteobateria* represented 87.6% of total OTUs (1601), of which *Betaproteobacteria* accounted for 51.2%, and *Gamma-* and *Alphaproteobacteri*a together represented 34.4%. In addition, *Bacteroidetes*,* Actinobacteria*, and *Planctomycete*s occurred in all sediments and in overlying water. Cluster analysis showed that bacterial community structures of SC and SG sediments were similar, but those of the SC overlying water were different than the sediment communities (Fig. 3).

The six places corrected are in bold type. The authors apologize for this and regret this oversight.

