# Supplementary material for: Evaluating bacterial community structures in oil collected from the sea surface and sediment in the northern Gulf of Mexico after the Deepwater Horizon oil spill
Source: Microbiologyopen. 2013 Apr 9;2(3):492–504. doi: 10.1002/mbo3.89 (PMC3684762; doi:10.1002/mbo3.89)
Supplement: Supplementary file 3 [file mbo30002-0492-SD3.docx]

Figure S1. Rarefaction curves of the samples collected in the northern Gulf of Mexico during or after the *Deepwater Horizon* oil spill. The samples included oil mousse collected at stations OSS, CT and MP. Ambient water without visible oil was also collected at station OSS and CT (OSS-W, CT-W). The overlying water was collected at station SC (SC-W). Rarefaction curves of all samples reached saturation, indicating that our sampling and sequencing efforts adequately covered the extent of taxonomic diversity at 95% confidence level.

Figure S2. Rarefaction curves of the sediment samples collected at stations SG and SC in the northern Gulf of Mexico one year after the *Deepwater Horizon* oil spill. Rarefaction curves of both samples reached saturation, indicating that our sampling and sequencing efforts adequately covered the extent of taxonomic diversity at 95% confidence level.

Table S1. Identification of known oil degraders in the *Gammaproteobacteria* class. The data showed both operational taxonomic units (OTUs) and the percentages of the bacterial species.

|  | ^#^OSS-W | CT-W | OSS | CT | MP | SG | SC | SC-OW |
| --- | --- | --- | --- | --- | --- | --- | --- | --- |
| **Legionellales** | 2 (0.03) | *-- | -- | -- | -- | 2 (0.3) | 7 (1.2) | -- |
| **Alteromonadales** | 1 (0.01) | -- | 453 (14.3) | 356 (17.9) | 215 (8.4) | 15 (2.2) | 41 (7.1) | 334 (10.7) |
| *Marinobacter* | -- | -- | 183 (5.8) | 298 (15.0) | 123 (4.8) | -- | 5 (0.9) | -- |
| *Shewanella* | 1 (0.01) | -- | -- | 17 (0.9) | 72 (2.8) | 2 (0.3) | 2 (0.4) | -- |
| *Alteromonas* | -- | -- | 259 (8.2) | 32 (1.6) | 1 (0.04) | -- | -- | -- |
| *Saccharophagus* | -- | -- | -- | -- | -- | -- | -- | 267 (8.5) |
| *Colwellia* | -- | -- | -- | -- | -- | 2 (0.3) | 8 (1.4) | -- |
| **Oceanospirillales** | -- | -- | 123 (3.9) | 76 (3.8) | 19 (0.7) | 4 (0.6) | 14 (2.4) | -- |
| *Alcanivorax* | -- | -- | 54 (1.7) | 56 (2.8) | 10 (0.4) | 1 (0.2) | 2 (0.4) |  |
| **Pseudomonadales** | -- | -- | 1 (0.03) | 96 (4.8) | 168 (6.5) | 33 (4.9) | 78 (13.5) | 6 (0.2) |
| *Pseudomonas* | -- | -- | 1 (0.03) | 69 (3.5) | 120 (4.7) | 33 (4.9) | 78 (13.5) | 6 (0.2) |
| **Thiotrichales** | -- | -- | -- | -- | -- | 2 (0.3) |  | 6 (0.2) |
| *Cycloclasticus* | -- | -- | -- | -- | -- |  |  | 4 (0.1) |
| **Chromatiales** | -- | -- | 78 (2.5) | 9 (0.5) | 2 (0.08) | 20 (3.0) | 6 (1.0) | 28 (0.9) |
| *Thiorhodovibrio* | -- | -- | 60 (1.9) | 7 (0.4) | -- | -- | -- | -- |
| **Vibrionales** | -- | -- | 10 (0.3) | 9 (0.4) | 1536 (59.7) | -- | -- | 114 (3.6) |
| *Vibrio* | -- | -- | 10 (0.3) | 9 (0.4) | 1535 (59.6) | -- | -- | 114 (3.6) |
| **Methylococcales** | -- | -- | -- | -- | -- | 22 (3.3) | 38 (6.6) | 172 (5.5) |
| *Methylobacter* | -- | -- | -- | -- | -- | 2 (0.30) | 13 (2.2) | 157 (5.0) |
| *Methylococcus* | -- | -- | -- | -- | -- | 15 (2.2) | 19 (3.3) | -- |
| **γ-proteobacteria (order)** | -- | -- | -- | -- | 8 (0.31) | 9 (1.4) | 10 (1.7) | -- |
| **Xanthomonadales** | -- | 2 (0.03) | -- | 4 (0.2) | -- | -- | -- | -- |
| **Salinisphaerales** | -- | -- | -- | -- | 3 (0.12) | 0 | -- | -- |
| **Aeromonadales** | -- | -- | -- | -- | -- | 2 (0.3) | -- | -- |

*: “--” means not detectable;

#: OSS-W and CT-W represent ambient surface waters sampled at stations OSS and CT. OSS, CT and MP refer to the oil mousse sampled at these stations. SG and SC were the oil-contaminated sediments adjacent to the wellhead. SC-OW was the overlying waters of the SC sediment.

Table S2. Identification of known oil degraders in the *Alphaproteobacteria* class. The data showed both operational taxonomic units (OTUs) and the percentages of the bacterial species.

|  | ^#^OSS-W | CT-W | OSS | CT | MP | SG | SC | SC-OW |
| --- | --- | --- | --- | --- | --- | --- | --- | --- |
| **Rhodobacterales** | 20 (0.3) | 69 (1.2) | 1436 (45.3) | 377 (19.0) | 297 (11.5) | 14 (2.1) | 24 (4.2) | 27 (0.9) |
| *Rhodovulum* | *-- | -- | 548 (17.3) | 16 (0.8) | -- | -- | -- | -- |
| *Stappia* | -- | -- | 204 (6.4) | 111 (5.6) | 15 (0.6) | -- | -- | -- |
| *Roseobacter* | -- | 2 (0.03) | 54 (1.7) | 18 (0.9) | -- | 4 (0.6) | 7 (1.2) | -- |
| *Paracoccus* | -- | 14 (0.2) | 100 (3.2) | 4 (0.2) | 5 (0.2) | 5 (0.8) | -- | -- |
| *Roseovarius* | -- | -- | 70 (2.2) | 53 (2.7) | 108 (4.2) | -- | -- | -- |
| *Oceanicola* | -- | -- | 104 (3.3) | 8 (0.4) | 2 (0.1) | -- | -- | -- |
| *Hyphomonas* | 4 (0.06) | 3 (0.05) | 76 (2.4) | 10 (0.5) | 4 (0.2) | -- | -- | -- |
| *Oceanicaulis* | -- | -- | 34 (1.1) | 76 (3.8) | 3 (0.1) | -- | -- | -- |
| *Ruegeria* | 2 (0.03) | -- | 44 (1.4) | 9 (0.4) | 3 (0.1) | -- | -- | -- |
| *Thalassobacter* | -- | -- | 42 (1.4) | 8 (0.4) | -- | -- | -- | -- |
| **Rhizobiales** | 69 (1.0) | 35 (0.6) | 435 (13.7) | 296 (14.9) | 14 (0.5) | 82 (12.3) | 35 (6.1) | -- |
| *Bartonella* | -- | -- | 293 (9.2) | 72 (3.6) | -- | -- | -- | -- |
| *Methylobacterium* | 2 (0.03) | 2 (0.03) | -- | 100 (5.0) | -- | -- | -- | -- |
| *Agrobacterium* | -- | -- | -- | 69 (3.5) | -- | -- | -- | -- |
| *Parvibaculum* | 32 (0.5) | 16 (0.3) | 45 (1.4) | 16 (0.8) | -- | 4 (0.6) | -- | -- |
| *Rhodobium* | -- | -- | 8 (0.2) | -- | -- | 23 (3.4) | 3 (0.5) | -- |
| *Prosthecomicrobium* | -- | -- | -- | -- | -- | 24 (3.59) | 2 (0.4) | -- |
| *Hyphomicrobium* | -- | 5 (0.1) | -- | -- | -- | 19 (2.8) | 21 (3.6) | -- |
| **Rhodospirillales** | 4 (0.1) | -- | 102 (3.2) | 521 (26.2) | 27 (1.0) | 73 (10.9) | 40 (6.9) | -- |
| *Thalassospira* | -- | -- | 32 (1.0) | 470 (23.7) | 26 (1.0) | 8 (1.2) | -- | -- |
| *Magnetospirillum* | -- | -- | 29 (0.9) | 13 (0.6) | -- | 2 (0.3) | -- | -- |
| *Rhodospirillum* | -- | -- | 1 (0.03) | 2 (0.1) | -- |  | -- | -- |
| *Rhodovibrio* | -- | -- | -- | -- | -- | 16 (2.40) | 15 (2.60) | -- |
| **Rickettsiales** | 1 (0.01) | 10 (0.17) | 1 (0.03) | -- | -- | -- | 2 (0.35) | -- |
| **Sphingomonadales** | 30 (0.4) | 17 (0.3) | 326 (10.3) | 50 (2.5) | 46 (1.8) | 2 (0.3) | 7 (1.2) | 53 (1.7) |
| *Erythrobacter* | -- | -- | 200 (6.3) | 24 (1.2) | 26 (1.0) | -- | -- | -- |
| *Sphingopyxis* | -- | -- | 26 (0.8) | 2 (0.1) | 2 (0.1) | -- | -- | -- |
| *Sphingomonas* | 17 (0.2) | 8 (0.1) | 55 (1.7) | 9 (0.4) | -- | -- | 5 (0.9) | 53 (1.7) |
| *Sphingobacterium* | 2 (0.03) | -- | 2 (0.06) | -- | -- | -- | -- | -- |
| *Sphingosinicella* | -- | -- | 18 (0.6) | 6 (0.3) | -- | -- | -- | -- |
| **Caulobacterales** | 6 (0.09) | 3 (0.05) | 47 (1.5) | 56 (2.8) | 17 (0.7) | -- | -- | 335 (10.7) |
| *Caulobacter* | 3 (0.04) | 1 (0.02) | 37 (1.2) | 44 (2.2) | 17 (0.7) | -- | -- |  |
| *Brevundimonas* | 3 (0.04) | 2 (0.03) | -- | 8 (0.4) | -- | -- | -- | 335 (10.7) |
| **Kordiimonadales** | -- | -- | 2 (0.06) | -- | -- | -- | -- | -- |
| **Parvularculales** | -- | -- | 27 (0.8) | -- | -- | -- | -- | -- |
| **Sneathiellales** | -- | -- | -- | -- | -- | 4 (0.6) | -- | -- |
| **Kiloniellales** | -- | -- | -- | --- | -- | -- | 2 (0.4) | -- |

*: “--” means not detectable; ^#^The sample information is the same as those described above.
